# Roles for cerebellum and subsumption architecture in central pattern generation

**DOI:** 10.1007/s00359-023-01634-w

**Published:** 2023-05-02

**Authors:** John C. Montgomery

**Affiliations:** Institute of Marine Science, University of Auckland, Auckland, New Zealand

**Keywords:** CPG, Error learning, Basis function, Adaptive filter

## Abstract

Within vertebrates, central pattern generators drive rhythmical behaviours, such as locomotion and ventilation. Their pattern generation is also influenced by sensory input and various forms of neuromodulation. These capabilities arose early in vertebrate evolution, preceding the evolution of the cerebellum in jawed vertebrates. This later evolution of the cerebellum is suggestive of subsumption architecture that adds functionality to a pre-existing network. From a central-pattern-generator perspective, what additional functionality might the cerebellum provide? The suggestion is that the adaptive filter capabilities of the cerebellum may be able to use error learning to appropriately repurpose pattern output. Examples may include head and eye stabilization during locomotion, song learning, and context-dependent alternation between learnt motor-control sequences.

## Introduction

As a perspective paper, this contribution aims to explore new ideas of how the cerebellum may interact with central pattern generators (CPGs) to enhance, and perhaps even transform, their output.

CPGs are neuronal networks that produce rhythmic activation of muscles. They are a fundamental construct within the vertebrate nervous system, from agnathan (jawless) lampreys through to humans. Lampreys, as an early representative of vertebrate evolution, have been a classic model for understanding the detailed structure and operation of vertebrate CPGs in swimming (Grillner and El Manira 2020). Across vertebrates, locomotion encompasses a wide operational range, from swimming to limb-based flight and bipedal locomotion, and it is generally accepted that CPGs are operational across this range (Klarner and Zehr 2018). It is also evident that swimming CPGs are largely understood, but that the operation of limb CPGs, particularly in bipedal locomotion, remains enigmatic (Grillner and Kozlov 2021).

Across the evolutionary spectrum of vertebrate locomotion, a number of other animal models have also been material to our understanding of CPGs [e.g., tadpoles (Sillar and Li 2020); turtles (Stein 2018); and quadrupedal mammals (Nishimaru and Kudo 2000)]. In addition to locomotion, CPGs are also instrumental in other behaviours, such as electric pulses in weakly electric fish (Borde et al. 2020); swallowing and respiration (Pitts et al. 2021), chewing (Widmer and Morris-Wiman 2018); and vocalization (Rosner et al. 2018; Kelley et al. 2020). It is also fair to say that in many of these other behaviours, the detailed operation/interaction of CPGs, sensory interaction, and other control contributions could also be described as enigmatic (for a review of some aspects of this complexity, see Montgomery and Perks 2019). The term ‘enigmatic’ essentially means that our understanding of how CPGs integrate with other neural networks, to execute all the behaviours outlined above, falls short of Marr’s criteria for understanding brain function (Marr 1982). These criteria include: (a) the process is defined and behaviourally characterized; (b) the relevant computational algorithm(s) are identified; and (c) there is a detailed understanding of how neurons and their networks execute the algorithm. The focus of this perspective piece is to briefly scan the ways in which CPG function is controlled and expanded by neuromodulation and sensory inputs to ask the question: What are the limits to these options, and what behavioural patterns might require additional subsumption control layers such as cerebellum?

**Fig. 1:**
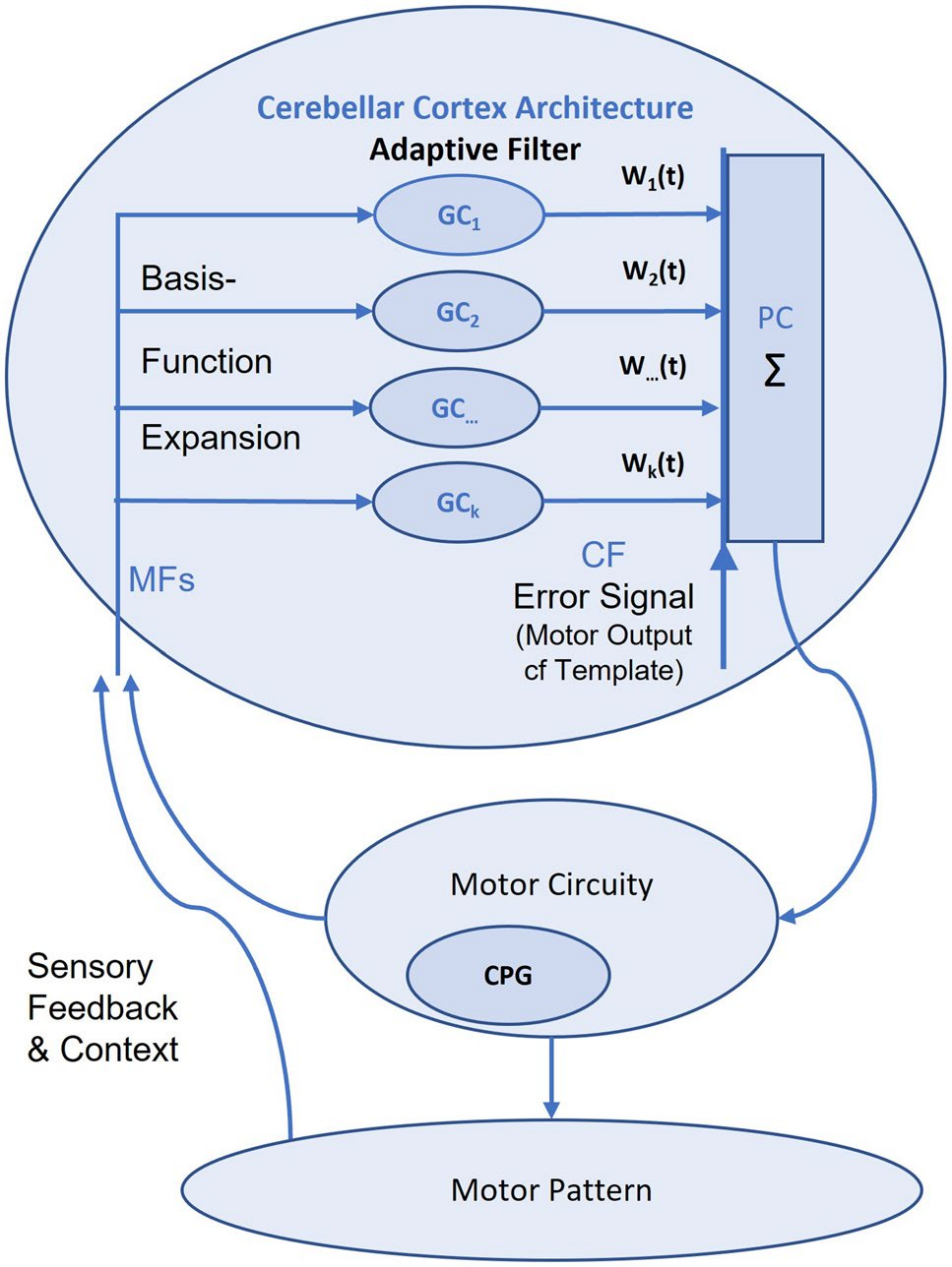
Schematic of cerebellar adaptive filter contribution to CPG pattern expansion. The cerebellar cortex receives input from mossy fibres (MFs) that terminate on granule cells (GC). From the adaptive filter perspective, the mossy fibre/granule cell pathway provides a basis-function expansion of input to the Purkinje cells (PC) via the parallel-fibre axons of the granule cells. The strength of the individual parallel-fibre inputs is a weighted input which varies with time (*W*_*i*_(*t*)), since the strength can be changed by a decorrelation learning rule when a parallel-fibre input occurs simultaneously with error signal input provided by climbing fibres (CF). From the CPG perspective, CPG output is directed to the adaptive filter where basis-function expansion extends the temporal availability of each output signal. The decorrelation learning rule at the parallel-fibre/Purkinje cell synapse adjusts the *W*_*i*_ as a function of time to decrease the error signal (which in the case of song learning is the difference between the motor output and the song template). Recurrent sensory feedback and/or context information can provide additional input to expand the pattern complexity, and/or make the output pattern context-appropriate. The term ‘tangled bank’ of adaptive filters refers to the massive bank of filters that make up the cerebellar cortex, and references Darwin’s ‘tangled bank’ metaphor for ecological complexity (Diagram after Porrill et al. 2013 and Montgomery and Perks 2019)

The cerebellum is a complex structure that has wide connections with many brain regions. To get a sense of the complexity of the cerebellum, in humans, it is credited with having 68 billion neurons (reviewed in von Bartheld et al. 2016), which corresponds to about 70% of the neurons in our brain. Therefore, we need to break things down to navigate a way through this complexity. One way is to look at the cerebellar cortex as a ‘tangled bank’ of adaptive filters (Fig. 1; Montgomery and Perks 2019; Dean et al. 2010). Each adaptive filter module is formed around a Purkinje cell (or small group of Purkinje cells). The Purkinje cell axons are the output pathway from the cerebellar cortex. Two very different inputs provide the functional architecture for the adaptive filter. First, the mossy fibre, granule cell, parallel-fibre pathway with associated interneurons. Axons of the granule cells form the parallel-fibre structure of the cortex and provide rich complex input to the Purkinje cell dendrites. The second input pathway is essentially the polar opposite: for each adaptive filter module, a single climbing fibre that forms multiple synaptic connections with the soma and basal dendrites of the Purkinje cell. From an adaptive filter perspective, the parallel fibres provide the basis-function input to the filter, and the climbing fibres error signals to appropriately adjust the filter output. From this standpoint, the cerebellum is effectively defined by its computational algorithm, and arguably there is a relatively good understanding of how the adaptive filter algorithm is executed by the cerebellar neurons and their networks. What is most needed to complete the picture is the way in which the cerebellum is connected to, and contributes to, defined and behaviorally characterized process. Or from a CPG perspective, what is the potential utility of incorporating adaptive filter functionality into CPG associated behavior?

An important consideration in exploring CPG/cerebellar interactions is the idea of subsumption architecture. This is borrowed from the software computing domain where it was originally proposed as an efficient way of adding functionality to a pre-existing network (Brooks 1986). Lampreys are endowed with multiple CPGs, in the hindbrain and down the length of the spinal cord, but essentially have no cerebellum. Their closest jawed vertebrate relatives are elasmobranchs, including sharks, which have extensive cerebellum-like structures in the dorso-lateral wall of the hindbrain, a distinct vestibulo-cerebellum, and well-developed corpus cerebellum. Indeed, in some shark species, cerebellum and cerebellum-like structures comprise over 50% of their brain volume (Yopak and Montgomery 2008). Clearly, lamprey have a functioning brain, so the advent of cerebellum as an additional system overlying this pre-existing functional network qualifies as subsumption architecture. It is also worth noting that the origin of these cerebellar structures in elasmobranchs sits alongside the evolutionary innovations of jaws, paired fins, and enhanced 3D mobility, which may in turn depend on cerebellar support. Furthermore, it has been suggested that the evolutionary innovation of an evolvable subsumption architecture could well be a factor in the subsequent extensive radiation of vertebrates (Yopak et al. 2010). Which in effect brings us back to the question: what are limits of the CPG functionality and flexibility without a cerebellum, and what might be gained from adding a tangled bank of adaptive filters? How might the cerebellum interact with CPGs to enhance, and perhaps even transform, their output?

Before addressing the questions above, it is worth noting that the biological literature on CPGs has been material in bioinspired robotic design (e.g., Ijspeert et al. 2007). There is a whole biomimetic research area, that not only benefits from a better understanding of biological movement control, but that can also interactively test and challenge that understanding. This particular perspective on CPG/cerebellar interactions is a venture into relatively uncharted territory with the aim of opening up new ideas and possibilities both for our understanding of CPGs and movement control, and also potential engineering applications.

Given that the cerebellum is a vertebrate construct, the focus of this article is on vertebrate brain and CPGs. However, it would be useful for non-vertebrate CPG experts to cross-check the proposed roles for the cerebellum. Finding this same functionality in invertebrate examples would argue against including a cerebellum subsumption layer as a necessary component—unless of course the invertebrate in question had a cerebellum-like neural network, as has been suggested for insects (Farris 2011) and octopus (Wang and Ragsdale 2019).

## The biological scope of vertebrate CPG motor behaviour without a cerebellum

In lamprey, tadpoles and larval zebrafish CPG control of swimming is well understood (Grillner and Kozlov 2021; Sillar and Li 2020). Each spinal segment has a CPG network on both sides, which are linked across the midline by inhibitory commissural interneurons. Intersegmental connections result in a rostro-caudal wave of activity that produces forward swimming. If the lead segment of activity is located caudally, this can reverse the activity wave to produce backward swimming. Tadpoles also generate a distinct form of backward swimming described as struggling. Descending drive from the hindbrain modulates the activation levels and CPG recruitment to initiate and control swimming speed (Grillner and Koslov 2021), and neuromodulation plays a role in adapting locomotor output to prevailing demands (Sillar and Li 2020). Proprioceptive sensory feedback is derived from body curvature and can directly influence swimming performance and efficiency (Hamlet et al. 2018). Lampreys have no cerebellum, and the extent to which cerebellum is developed and/or operational in tadpoles and larval zebrafish swimming is undetermined.

Spinal cord CPGs increase in complexity and functionality across the range of vertebrate species. For example, CPGs in the turtle spinal cord exhibit a range of computational complexity even in the absence of descending connections. These include three forms of scratch, two forms of swim, and one form of flexion reflex, and two further CPG patterns (terrestrial and bottom walking) that have yet to be studied (Stein 2018; Bannatyne et al. 2020). Although anecdotal: with respect to walking and swimming, it is remarkable to watch newly hatched turtles, as a number of us did at Heron Island (at the Sensory Processing of the Aquatic Environment meeting 1999). To see them emerge from the sand, with hungry gulls all around, orient and quickly move down the beach to the water’s edge. Once in the water, the asymmetrical walking gait quickly switches to symmetrical swimming and the newly hatched turtles disappear out to sea, reportedly to clear the reef before the diurnal predators awake. Although a role for the cerebellum in this behaviour has not been ruled out, it seems unlikely given the immediate post-hatching and predetermined nature of the behaviour and CPG transition.

The operation of CPG in an isolated spinal cord provides clear examples of possible vertebrate CPG motor behaviour without a cerebellum. Shifting to CPG in the hindbrain, the exclusion of a cerebellar contribution to the behaviour becomes less clear. Being mindful that ‘absence of evidence is not evidence of absence’, the following examples still seem to fit within the category of the biological scope of vertebrate CPG behaviour without a cerebellum.

The electric organ discharge (EOD) of weakly electric fish is a rhythmic and stereotyped electromotor pattern and is said to be controlled by a hindbrain CPG; however, the term ‘pacemaker’ nucleus (PN) may be more appropriate. The EOD is used as the basis for an active electrosense and differs across species from pulse type discharges to high-precision stereotypic waveforms (Shifman et al. 2020). Therefore, the PN differs from most CPGs with respect to generating single pulses and/or higher cycle rate, and also simpler pattern output when compared with locomotion, and other CPG generated patterns. However, in common with CPGs, EOD also exhibits within-species functional versatility across maturation state and social context that have been shown to be controlled by pre-pacemaker influences and hormonal modulation (Borde et al. 2020).

Vocalization is another hindbrain CPG generated motor pattern that has been extensively studied in fish and amphibian models. Both toadfish, and frogs, are capable of exhibiting multiple classes of social context-dependent vocalizations with widely divergent temporal and spectral properties. In toadfish, the source of pattern versatility is thought to be pre-pacemaker nuclei, and other modulatory inputs from neurons adjacent to vocal CPG neurons (Rosner et al. 2018). In Xenopus, vocal signals differ between the sexes, through development, and across different species within the genus. The hindbrain vocal CPGs generate species-specific vocal patterns based on their intrinsic properties, vocal nuclei connections, and neuromodulation (Kelley et al. 2020).

## Cerebellum examples relevant to CPG/cerebellar interaction

Before directly addressing potential CPG/cerebellar interactions, it is useful to summarize some relevant examples of cerebellar contributions to behaviour, and to gain a sense of cerebellar changes across vertebrate evolution.

Cerebellum-like structures found in the dorso-lateral wall of the hindbrain are found in agnathans, elasmobranchs, bony fish, and amphibians (Boord and McCormick 1984), and their function is described in Bell et al. (1997) and Montgomery and Bodznick (2016). They are directly innervated by afferents of electrosensory and mechanosensory systems. Given their name, it is not surprising that their cortical structure is very similar to that of the cerebellum. The main difference is that the principal cells of these nuclei receive direct sensory input and are the main ascending efferent neurons (AENs) from these nuclei to the midbrain. The dorsal dendrites of the AENs are very like those of Purkinje cells and receive parallel fibre and other interneuron input. As with the cerebellum, the cortical parallel fibres are the axons of granule cells. The key advantage of understanding the function of these structures over that of cerebellum is that cerebellum-like structures receive and process direct sensory information. Both electro- and mechano-sensors detect and encode biologically important information, but both receptor types are very susceptible to activation from the animal’s own electric fields and movement (sensory reafference). Afferents convey both biologically relevant stimuli and sensory reafference to the AENs. The function of the AEN circuitry is to cancel the reafference, such that the output of the AEN transmits only the biologically relevant stimuli to higher brain centres. A significant part of this cancelation comes from the cortical structure. Granule cells receive multiple inputs, but from our perspective of CPG/cerebellar interactions, one of the most important is CPG efference copy for ventilation and movement. Synaptic plasticity between the parallel fibres and the AENs provides a decorrelation learning rule, such that AEN firing reduces the strength of concurrently active parallel fibres. The upshot is a forward model that can anticipate and cancel movement-related reafference. In effect, the cerebellum-like structures act as noise canceling headphones. Cerebellum-like structures are clear working example of the adaptive filter. Not in the main sense we are looking for: of the potential utility of incorporating adaptive filter functionality into CPG-associated behaviour; but sense of they do demonstrate the utility of plugging CPG efference copy into an adaptive filter to execute a noise cancelation mechanism. As is evident in this system, electrophysiological interrogation of the underlying mechanism depends on being able to make the neural recordings, while the animal is ventilating. The difficulties of making recordings during active movement have meant that this approach is relatively understudied.

The second example is a brief summary of the role of cerebellum in vestibulo-ocular reflex (VOR) gain control as an exemplar of subsumption architecture. Lampreys have an operational brain with effectively no vestibulo- or corpus cerebellum. They have an operational VOR, but no evidence of active gain control equivalent to the that found in other vertebrates (Wibble et al. 2022). As previously said, the advent of cerebellar structures in elasmobranchs, as an additional network overlying a pre-existing functional brain, qualifies as subsumption architecture. Focusing on the vestibulo-cerebellum, this plays a key role in tuning the open-loop VOR. The function of the VOR is to maintain eye/visual stability during head movement. In some ways, it is a mechanical analogue of sensory noise cancelation. The vestibular system senses head movement and activates secondary vestibular neurons in the hindbrain, which in turn activate ocular motor neurons to counter-rotate the eye, stabilizing the visual image of the external world. If the VOR is underperforming, the resulting visual slip error signal is used by the vestibulo-cerebellum to intervene and adjust the VOR gain. Direct vestibular information goes to the vestibulo-cerebellum through the granule cell/parallel fibre pathway. The Pukinje cells project directly back to the secondary vestibular neurons. Visual slip error signals conveyed by climbing fibres adjust the Purkinje cell output to correct the VOR and restore appropriate function. This overlying vestibulo-cerebellar functionality would qualify as subsumption architecture in its own right; however, in this case, subsumption control goes one level further. Through time, the Purkinje cell activity readjusts the strength of vestibular afferent/secondary neuron connections to tune the correct VOR gain, so the vestibulo-cerebellar contribution is no longer required. With the gain of the VOR system corrected, there is no longer any need for active participation of the cerebellar subsumption network (see Montgomery and Bodznick 2016 for further detail).

The cerebellum-like structures in the fish brain provide a forward model of sensory consequences of active movement. It should be noted that in ‘noise cancellation’, the forward model is a sculptured derivative of the CPG efference copy which is different from corollary discharge gating. This distinction is recognized by Straka et al. (2018), but also with the caveat that there are difficulties in making a clear functional separation between the two. These considerations also apply to the widespread way in which the mammalian brain deals with the sensory consequences of movement through forward-model cancelation and/or gating (e.g., Kilteni and Ehrsson 2022; Mackrous et al. 2022; Lambert et al. 2012). For forward-model cancelation, efference copy signals from the motor-control systems are thought to be processed by the cerebellum to provide the expectation of the sensory consequences of the movement. These expectations may be processed in a variety of ways. Forward model expectations can be used in the suppression of sensory feedback (not dissimilar to the cerebellum-like noise cancelation). One of the best studied examples of this is the attenuation of self-tickle (Blakemore et al. 1999; Boehme and Olausson 2022). Experimental studies with humans show that as the tickling movement is progressively disassociated with the self-tickle movement, the perception of tickle becomes stronger. Second, where there is a mismatch between expectation and the actual eventuality, this can be used as error signals for adaptive motor control. Third, there are also good examples of where the expectations translate to adaptive learning to stabilize the body from perturbations caused by our own active movement. One of the best examples is the ‘off-loading’ response (Diedrichsen et al. 2003). If you are holding, say, a glass of beer in one hand, and you ask someone else to take it from you, you are unable to stabilize your hand the off-loading response. If, however, you take the glass with your other hand, your first hand remains stable throughout the transaction. Interestingly, the same thing happens when the weight is being removed by a robot. The off-loading response remains until you are given the opportunity to trigger the robotic off-load. Once you have specific timing information, you are then able to progressively learn to stabilize the hand. One of the important points to note here is that during the self-generated off-load, there is a wealth of motor command efference copy, whereas this is quite sparce in the case of triggering the robot. The speculation is that basis-function expansion in the parallel-fibre/granule cell system extends the dynamic information to allow the appropriate learning for the hand stabilization.

The concept of basis-function expansion is crucial to understanding the potential role of cerebellum and its potential interact with CPGs (Fig. 1). The case for basis-function expansion is spelt out in Montgomery and Perks (2019). The summary points of evidence for this case are as follows:

1. The huge number of granule cells in human cerebellum, with 200+ times more granule cells than mossy fibres. This clearly indicates that information is not just relayed by granule cells, but that there is a processing/expansion of information at the mossy fibre/granule cell interface.

2. The importance and scope of basis-function expansion are well demonstrated in the cerebellar model system of delay conditioning. This is an important learning paradigm that explicitly demonstrates basis-function expansion and cerebellar adaptive filter functionality. In this paradigm, an air-puff to the eye produces an eye blink (think the current methodology for doing intraocular pressures). If the air-puff is preceded by a tone, then the subject learns to blink just prior to the air-puff presentation. In associative learning lexicon, the air-puff is the unconditioned stimulus, and the blink is the unconditioned response. The tone is the conditioning stimulus and the predictive eyeblink is the conditioned response. After learning, if the cerebellum is deactivated, the conditioned response still occurs but is triggered by the tone onset, and no longer timed to anticipate the air-puff. One interpretation consistent with experimental findings is that basis-function expansion diversifies and re-codes the tone from an analogue step function to a burst sequence across a population of granule cells. This would provide sequential information for Purkinje cell inhibition of the blink until the appropriate time at which point a pause in inhibition would cause the blink.

3. Burst firing of granule cells is seen in individual cells (Marín et al. 2020), but sequential burst firing as suggested by Bratby et al. (2017) would not show up in individual granule cell patch-clamp recordings. Recent developments in the applications of two photon microscopy are consistent with burst firing of individual granule cells and may be able to test the sequential burst firing hypothesis (Lanore et al. 2021). It would be useful to apply this methodology to investigate granule cell encoding of a step-function tone stimulus. If this burst sequence coding is verified, then the underlying mechanism becomes an intriguing question. The biologically plausible mechanism for the generation of predictable sequences of parallel fibre activity suggested by Bratby et al. (2017) implements a modified form of winner-take-all, in which the level of inhibition generated by the ‘winner’ diminishes with time. It is this feature which enables the network to generate sequential patterns of activity, as each cell takes its turn to outcompete its neighbours. Gaining a full biological understanding of a network of billions of granule/golgi cells, how they self-connect and the underlying plasticity rules that operationalize the network is an intriguing, but difficult, challenge, but one that could well have useful biomimetic application.

4. Human delay conditioning typically operates in the range of 500–800 ms but does extend up into the 1000–1350 ms range (Cheng et al. 2008; Caulfield et al. 2015; Herbert et al. 2003). By comparison, typical walking cadence equates to about 500 ms/step cycle, breathing cycle to about 4000 ms, and a single line of a nursery rhyme somewhere in between these. Meaning that the basis-function expansion evident in delay conditioning could also apply to the total CPG cycle for walking, and a significant part of other slightly longer CPG cycles.

To summarize this section. CPG efference copy has been demonstrated to be a major contribution to cerebellum-like structures involved in forward model cancelation of sensory reafference. Vestibo- and corpus-cerebellum structures evolved over the top of a functioning brain, and exhibit elements of subsumption architecture. CPG efference copy input to cerebellum can play a role in forward model expectations used in the suppression of sensory feedback, error signals for adaptive motor control, and adaptive learning to stabilize the body from perturbations caused by active movement. Finally, proliferation of granule cells is a prominent feature of cerebellum evolution and ensuing basis-function expansion an important candidate for cerebellum interactions with CPGs.

## CPG-related behavioural patterns that might require, and use, a cerebellar subsumption contribution

The first example refers back to VOR stabilization of the eyes during passive head movement. Recent work on tadpoles (Straka et al. 2022) that has also recently been generalized to mice (de Barros et al. 2022) shows that during active movement, the motor-control signals are available to provide an alternate means of eye stabilization. The important distinction here is the difference between active and passive movement which as Straka et al. (2018) say provides “a new perspective on predictive motor signaling”. The distinction between active and passive movement is also important from an experimental perspective. As with sensory reafference studies, the vast majority of experimental interrogations of the neural mechanisms of movement control have not been done during active movement. The tadpole model explicitly shows that during active swimming, the passive VOR is suppressed, and the eye stabilization is driven from locomotion CPGs. However, the relative contributions of passive vs. active VOR do change through development (Bacqué-Cazenave et al. 2022). It is interesting to note that active control is seen in fictive locomotion in a hindbrain/spinal cord preparation, showing that the cerebellum is not required as a direct component of this control. However, as with passive VOR, it is entirely possible that the cerebellum may be able to adaptively moderate the CPG/eye stabilization circuitry, but that its subsumption capabilities may mean it only intervenes when required and then instantiates the adaptive correction into the underlying circuity. Therefore, this “new perspective on predictive motor signaling” resonates strongly with the premise of this perspective article, but in this system, we still lack direct evidence for a cerebellar role to enhance and/or transform, CPG output. Conceptually, it should be possible to test the role for a cerebellar contribution to the CPG by interrogating the neuronal integration of vestibulo- and spino-ocular reflex components during some form of gain change paradigm (such as, for example, in Dietrich and Straka 2016), perhaps at different frequencies and different developmental stages.

Before citing some examples of direct evidence for cerebellar involvement, it is worth looking some other examples of potential CPG/cerebellar movement control.

**Fig. 2:**
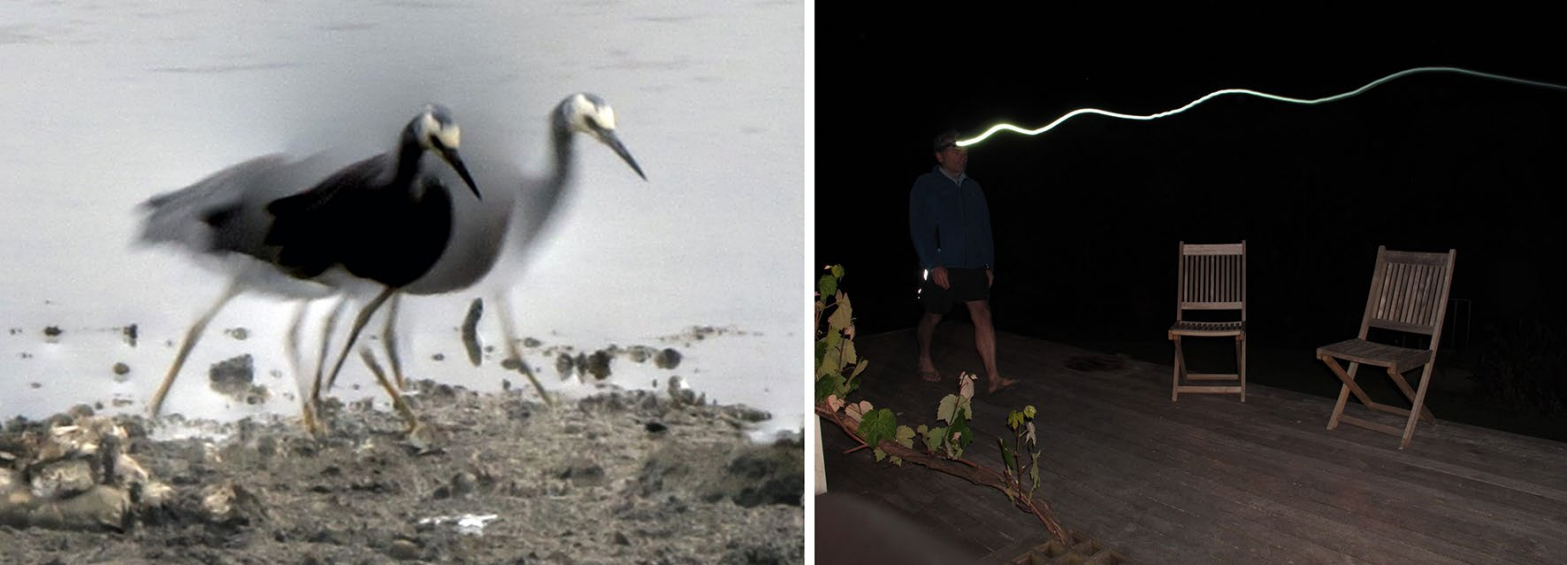
Head stabilization in walking heron and vertical head movement in human walking. **a** Head stabilization during walking in the white-faced heron (*Egretta novaehollandiae*). Four consecutive individual photos (0.25s exposure) of a walking heron automatically stacked in photoshop. The continuous movement of the body is evident and the two static positions of the head. The first frame to the left shows the blurred movement of the body with the head already positioned where it stays until the second frame. In the third frame, the blurred body is visible, but the head is in effect invisible as it is ‘saccading’ to the next position, where it is held for the fourth frame. **b** Flash and then long-exposure photograph showing vertical movement of the head (headlamp) during walking. Photographs J. Montgomery

In our book “Evolution of the Cerebellar Sense of Self” (Montgomery and Bodznick 2016), we suggest that head stabilization during walking in birds (Fig. 2a) would be a clear candidate for walking CPG-driven movement control. This head bobbing (Necker 2007), or “head nystagmus”, with the head held stationary during walking and then “saccading” to a new position also has the characteristic that during the stationary phase, there would be little or no vestibular or visual feedback.

**Fig. 3:**
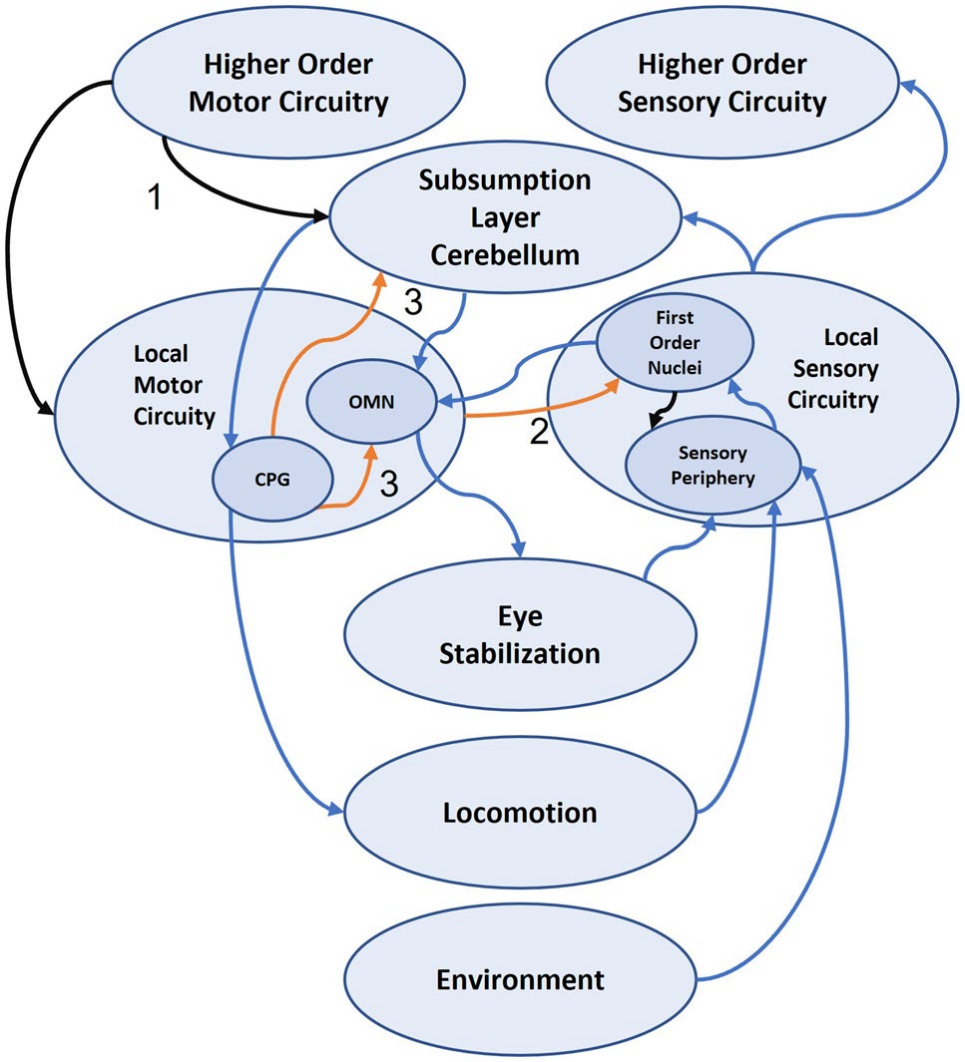
Suggested connectivity for vertical eye movement stabilization during walking based on Straka et al.'s (2018) template. Diagram provides a potential candidate for optokinetic/vestibulo-ocular reflex suppression and CPG-driven eye stabilization, with the added complexity of focal-point based gain control and cerebellar supervision of CPG oculomotor interaction. **1** Focal-point information contributes to eye stabilization gain either directly, or through cerebellar influence. **2** CPG efference copy to vestibular nuclei may activate vestibular efferents to gate sensory input, and/or alter the gain of the VOR pathway. **3** CPG efference copy may drive the oculomotor neurons (OMN) directly, and/or via the cerebellum to drive eye stabilization. Cerebellar influence may be on-line, or via learnt modification of the CPG OMN pathway through error learning

Walking in humans also produces vertical head movement (Fig. 2b), with associated vertical eye stabilization. When walking adjacent to a fence at eyelevel, demonstrate for yourself that the eye stabilization is focus dependent: focus on the fence top and this appears stationary with the background bouncing up and down; focus on the background and the alternate happens. This seems like another potential candidate for optokinetic/vestibulo-ocular reflex suppression and CPG-driven eye stabilization, with the added complexity of focal-point based gain control (Fig. 3). The recent reports of activity-dependent suppression of vestibular balance control (Dietrich et al. 2020), and down-beat nystagmus attenuation during walking would align with this view (Dietrich et al. 2022). Direct evidence for the coordination of gaze behaviours and walking have been shown in cats (Zubair et al. 2019), who argue that “gaze–stride coordination arises as a part of the whole-body locomotor synergy, which includes the limbs, body, head, and eyes.”

Vocal learning is a complex behaviour found in humans but elsewhere sparingly in mammals and extensively in birds. Songbirds and parrots make up over 60% of avian species. The neuroethology of birdsong has been extensively studied, as one of the model systems in this field. It is a rare example of a high-level motor control where the underlying components can be directly interrogated and are accessible to manipulative experiments at a cellular, synaptic, and organismal level. Furthermore, the neuroethology differences between learners and non-learners can be characterized. Song learners are distinctive with respect to song development period, dependence on learning templates, and specialized forebrain nuclei. The contribution of cerebellum to vocal learning in songbirds is evident in the anatomical connections between cerebellum and vocal centres, but functionally is still something of a smoking gun. It has been long proposed (see Doupe and Kuhl 1999, for a review), and the case recently made that there could be a distinctive role for the cerebellum as a massive array of adaptive filters to interact with CPGs in the construction and potentially modification of learnt song (Montgomery and Perks 2019). Vocal learning may, of itself, be diagnostic of a cerebellar contribution, but the demonstration that song leaning can be context-specific (Viet et al. 2021) further strengthens the case for cerebellar involvement. In the Viet et al.'s (2021) study, context-specific changes in both the pitch and sequencing of song were successfully induced and apparent even on single probe trials. Context-dependent alternation between learnt motor control sequences system may be an exemplar of the potential utility of incorporating cerebellar adaptive filter functionality into CPG-associated behaviour. The mechanism proposed for song learning by Montgomery and Perks (2019) is that CPGs (presumably allied to, and/or evolved from ventilation CPGs) send their output to the cerebellum. Basis-function expansion in the mossy fibre/granule cell network effectively allows temporal extension of each element of the CPG efference copy. Comparison with the external song template creates error information to allow the adaptive filter to learn the appropriate song pattern. Recurrent feedback of the output from a single CPG cycle could result in successive sequential pattern formation. This same mechanism could potentially enable learning of multiple context-dependent patterns.

Attempting to translate bird song to human vocal learning is complex (Zhang and Ghazanfar 2020). Obviously, bird song would have more in common with singing than speech, and there is reasonable evidence that human song and speech have differing underlying neural substrates. Speech is a complex and more deliberative behaviour. Apraxia of speech is a neurologic speech disorder, often associated with left-hemisphere cerebrovascular damage, with an impaired capacity to plan or program sensorimotor commands necessary for directing movements that result in normal speech (Henderson et al. 2019). The proposed mechanism for birdsong learning (above) is more mechanistic and could parallel neural mechanisms of human song learning. This connection aligns with the observation that many individuals with apraxia can sing the words of a song better than they can say the same words in a conversation (Henderson et al. 2019). Furthermore, people with aphasia (the inability to use and understand language) more accurately retrieve the lyrics of familiar songs when they are provided with the first half of a phrase in a sung version compared with a spoken version (Van Lancker Sidtis et al. 2021). It would be useful if there was better understanding of the relationship between cerebellar injury and song. However, when compared with the cerebral cortex there does seem to be a ‘cognitive’ bias in available information and interpretation, for example, in a recent chapter on the “brain mechanisms underlying singing” (Cohen et al. 2020), cerebellum barely gets a mention.

If we take the context-dependent alternation between learnt motor control sequence as the ‘gold(finch) standard’ of CPG/cerebellar interaction, it is useful to ask the question what human examples are there of analogous learnt motor control sequences that are demonstrably context-dependent? One example might be the alternate motor program triggered by sensory feedback from the interruption to normal walking caused by a trip or slip. For example, trip recovery training on a modified treadmill has been shown to have beneficial effects on actual trip recovery (Bieryla et al. 2007).

At a more general level, human cerebellar pathology is linked with deficits that might also relate to CPG/cerebellar interaction, such as ataxia that affects gait, posture, and patterns of movement (O’Sullivan et al. 2014). It is also interesting to see that in a recent review of the “Functional outcomes of cerebellar malformation”, Gill and Sillitoe (2019) include reference to the evolutionary origins of the cerebellum (Montgomery et al. 2012) and patterns of brain scaling across vertebrates (Yopak et al. 2010). This pattern of brain scaling refers to the hyperallometry of the telencephalon and cerebellum with respect to the rest of the brain, such that brains large in absolute size become more and more composed of these two structures. This evolutionary perspective includes the speculation that evolution of the cerebellum and its co-evolution with neocortex has been a permissive step for the evolution of complex behaviour, including CPG/cerebellar/cortical pattern generation. Or to quote Galliano and De Zeeuw (2014) is may well be “…time to take a step back from the serial exploitation of the reductionist potential of the scientific method and attempt a more careful synthesis, which takes into account the various components of the puzzle and does not neglect the ethological and evolutionary dimension of the research”.

Finally, arguably, the best examples of direct experimental evidence for cerebellar involvement in reprogramming CPG generated behaviour include the observation that mice without a functional cerebellum have close to normal limb locomotion, but with distinct deficits in head and tail stability (Machado et al. 2015). Furthermore, when limb locomotion itself is challenged, results show that split-belt adaptation of gait specifically depends on the cerebellum (Darmohrayet al. 2019). Anatomical pathways related to these findings include the demonstration that cerebellospinal neurons from the fastigial and interpositus deep cerebellar nuclei target pre-motor circuits in the ventral spinal cord and the brain and are required for skilled forelimb performance and skilled locomotor learning (Sathyamurthy et al. 2020).

## Conclusion

Central pattern generators are entities in their own right, but also clearly modulated by sensory feedback, descending control, and neuromodulation. This perspective attempts to set out a template role for additional subsumption architecture and the possible role of the cerebellum to enhance, or perhaps even transform, CPG output. The essential elements of the template are efference copy of motor control and CPG output to the cerebellum; basis-function extension of efference copy; and error learning to appropriately repurpose the CPG output. Given the complexity of motor control, and the real-world competence of animal locomotion, there is clearly a virtuous feedback loop between biological understanding, modelling, and engineering. From an engineering perspective, it would be interesting to see if this CPG/subsumption architecture provides additional functional utility. One of the advantages of engineering options is that elements such as basis-function extension could be built with simple tapped delay lines, which do not seem possible in the biological context. Although it is also worth considering that, given the biological systems are operating with billions of complex entities, future insights from biological understanding of self-connecting/optimizing networks, and details like matching pulse inputs to adaptive filter learning rules, could also prove to be of value to engineering application.

## Data Availability

No data was used in the article.
